# Evolutionary paths of streptococcal and staphylococcal superantigens

**DOI:** 10.1186/1471-2164-13-404

**Published:** 2012-08-17

**Authors:** Kayo Okumura, Yumi Shimomura, Somay Yamagata Murayama, Junji Yagi, Kimiko Ubukata, Teruo Kirikae, Tohru Miyoshi-Akiyama

**Affiliations:** 1 Department of Infectious Diseases, National Center for Global Health and Medicine, 1-21-1, Shinjuku-ku, Tokyo, 162-8655, Japan; 2 Graduate School of Infection Control Science, Kitasato University, 5-9-1, Shirokane, Minato-ku, Tokyo, 108–8641, Japan; 3 Department of Microbiology and Immunology, Tokyo Women’s Medical University, 8-1 Kawada-cho, Shinjuku-ku, Tokyo, 162-8666, Japan; 4 Department of Animal and Food Hygiene, Obihiro University of Agriculture and Veterinary Medicine, Inada-cho, Obihiro, Hokkaido, 080-8555, Japan; 5 Present affiliation: College of Pharmacy, Nihon University, Narashinodai, Funabashi, Chiba, 274-8555, Japan

**Keywords:** Superantigen, *Streptococcus*, *Staphylococcus*, Fenome comparison, Bayes MCMC

## Abstract

**Background:**

*Streptococcus pyogenes* (GAS) harbors several superantigens (SAgs) in the prophage region of its genome, although *speG* and *smez* are not located in this region. The diversity of SAgs is thought to arise during horizontal transfer, but their evolutionary pathways have not yet been determined. We recently completed sequencing the entire genome of *S. dysgalactiae* subsp. *equisimilis* (SDSE), the closest relative of GAS. Although *speG* is the only SAg gene of SDSE, *speG* was present in only 50% of clinical SDSE strains and *smez* in none. In this study, we analyzed the evolutionary paths of streptococcal and staphylococcal SAgs.

**Results:**

We compared the sequences of the 12–60 kb *speG* regions of nine SDSE strains, five *speG*^+^ and four *speG*^*–*^. We found that the synteny of this region was highly conserved, whether or not the *speG* gene was present. Synteny analyses based on genome-wide comparisons of GAS and SDSE indicated that *speG* is the direct descendant of a common ancestor of streptococcal SAgs, whereas *smez* was deleted from SDSE after SDSE and GAS split from a common ancestor. Cumulative nucleotide skew analysis of SDSE genomes suggested that *speG* was located outside segments of steeper slopes than the stable region in the genome, whereas the region flanking *smez* was unstable, as expected from the results of GAS. We also detected a previously undescribed staphylococcal SAg gene, *selW*, and a staphylococcal SAg -like gene, *ssl*, in the core genomes of all *Staphylococcus aureus* strains sequenced. Amino acid substitution analyses, based on dN/dS window analysis of the products encoded by *speG*, *selW* and *ssl* suggested that all three genes have been subjected to strong positive selection. Evolutionary analysis based on the Bayesian Markov chain Monte Carlo method showed that each clade included at least one direct descendant.

**Conclusions:**

Our findings reveal a plausible model for the comprehensive evolutionary pathway of streptococcal and staphylococcal SAgs.

## Background

Bacterial superantigens (SAgs) have been shown to cause the massive activation of host T cells, strongly influencing immunological disorders. To date, nearly 50 bacterial SAgs and related molecules have been described, primarily from Gram-positive bacteria [1-3]. *Streptococcus pyogenes* (GAS) is one species of bacteria that harbors SAg genes. Analyses of the entire genomes of 13 GAS isolates have shown that each contains two to seven SAg genes (Additional file 1), almost all located in the prophage regions of the genome. In contrast, genes encoding the SAgs *speG* and *smez* in GAS strains are not located on these mobile genetic elements, although some are surrounded by transposons. Thus, *speG* and *smez* in GAS may have been inherited from an ancestor by horizontal gene transfer. Although *speJ* in M1 GAS is not located on these mobile genetic elements, *speJ* is not conserved in the genome sequence of other GAS isolates, except for MGAS6180 (data not shown); in some strains, an SAg similar to *speC* is called *speJ*. We recently sequenced the entire genome of *Streptococcus dysgalactiae* subsp. *equisimilis* (SDSE) [DDBJ: AP010935] [4], a bacterium that causes life-threatening infectious diseases, including sepsis and streptococcal toxic shock syndrome, similar to GAS [5-7]. Analyses of its sequence showed that SDSE is the closest relative of GAS sequenced to date, with 65% identity (Additional file 2). Streptococcal bacteria other than GAS, such as *S. dysgalactiae* subsp. *dysgalactiae*[8] and *S. equi*[9-11], have been reported to harbor more than one gene encoding proteins similar to SAgs. In contrast, targeted microarray analyses of 216 GAS virulence genes including SAgs in 58 SDSE strains isolated from human infections showed that the only SAg gene present in SDSE was *speG*[12], with about 50% of SDSE strains not harboring this gene [13-15].

Other representative bacterial SAgs and their related products have been identified in *Staphylococcus aureus*[2]. At least 20 distinct staphylococcal SAgs have been described, including toxic shock syndrome toxin-1 (TSST-1), staphylococcal enterotoxins (SEs), and staphylococcal superantigen-like proteins (SSL), also called staphylococcal enterotoxin-like proteins (SEls) [1-3]. Almost all staphylococcal SAg genes are located in mobile genetic elements, such as prophages, transposons, plasmids, and pathogenicity islands (PIs). The distribution of these mobile elements among *S*. *aureus* isolates varies considerably [16]. PIs that harbor the gene encoding TSST-1 can be excised and transduced with high efficiency by a staphylococcal phage [17].

In addition to these staphylococcal SAgs, recent studies have identified staphylococcal superantigen-like proteins (SSLs, also known as SETs), which have structural features similar to those of SAgs but do not possess SAg activity [18]. All of the SSLs described to date are located in mobile genetic elements [2]. Interestingly TSST-1, a functional SAg, shows higher sequence and structural similarity to SSL than to staphylococcal SAgs [18].

Structural analysis of SAgs has suggested that they evolved through the recombination of two smaller β-strand motifs, similar to the immunoglobulin binding motifs of streptococcal proteins G and L and the oligosaccharide/oligonucleotide binding family, such as the B subunits of AB(5) heat-labile enterotoxins, including cholera toxin, pertussis toxin, and verotoxin [19,20]. However, the origin and evolutionary pathways of streptococcal and staphylococcal SAgs have not been well described.

To elucidate the origin of streptococcal SAgs based on *de novo* sequencing of SDSE strains and whole genome sequences, we have analyzed the synteny of the regions surrounding *speG* and *smez* in 13 GAS and 9 SDSE genomes. We also analyzed the genomic structures of all *S. aureus* strains for which whole genome data are available. We detected a previously undescribed gene that encodes a SEA-like protein (designated *selW*[21]) and genes encoding SSL-like proteins, all of which are conserved in all *S*. *aureus* strains sequenced to date and are located in the core chromosome, not in any mobile elements. These findings, in addition to amino acid substitution analyses based on window analysis, cumulative TA-skew analysis and evolutionary analysis according to the Bayesian Markov chain Monte Carlo method, which allows the evolutionary path of SAg to be determined in chronological order, we were able to trace the origin and molecular evolution of streptococcal and staphylococcal SAgs.

## Results and discussion

### Comparison of sequences of *speG* regions in the GAS and SDSE genomes

**Figure 1:**
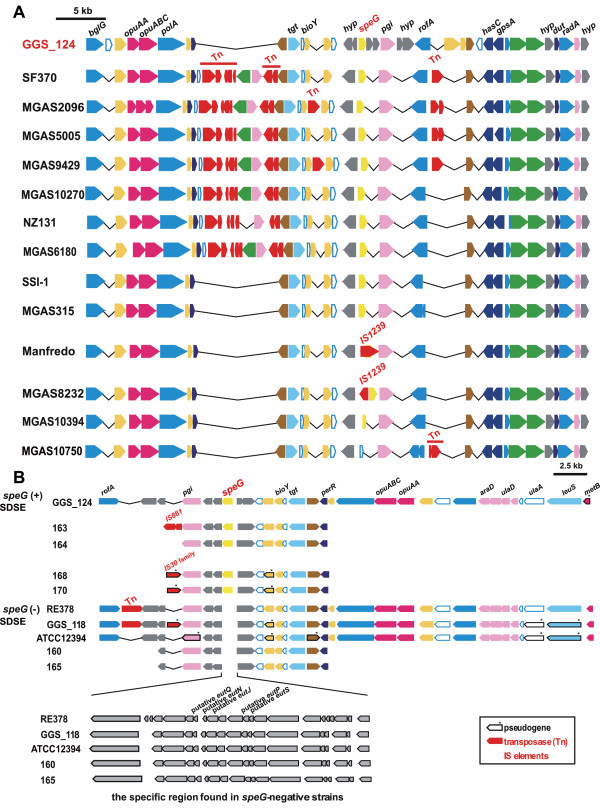
**Synteny mapping of *speG* regions in SDSE and GAS genomes.** (**A**) Genome context of *speG* and the 50 kb surrounding regions of the GGS_124 and 13 GAS strains. Each position (bp) on each genome is shown in Additional file 10. Some sequences encoding small peptides (20 to 30 amino acid residues) were annotated as having unknown functions or as hypothetical proteins and were omitted from this figure, because their assignments depended on the annotator. Transposase and IS elements are shown in red. *hyp* (in grey) indicates sequences encoding ‘hypothetical proteins’. Genes of the *speG* region of GGS_124 were inversely aligned. Pseudogenes are marked with asterisks. **(B**) Genome context of *speG* or the corresponding region in *speG*(+) and *speG*(−) SDSE strains**.** All information on the strains used in this study is shown in Additional file 1.

**Figure 2:**
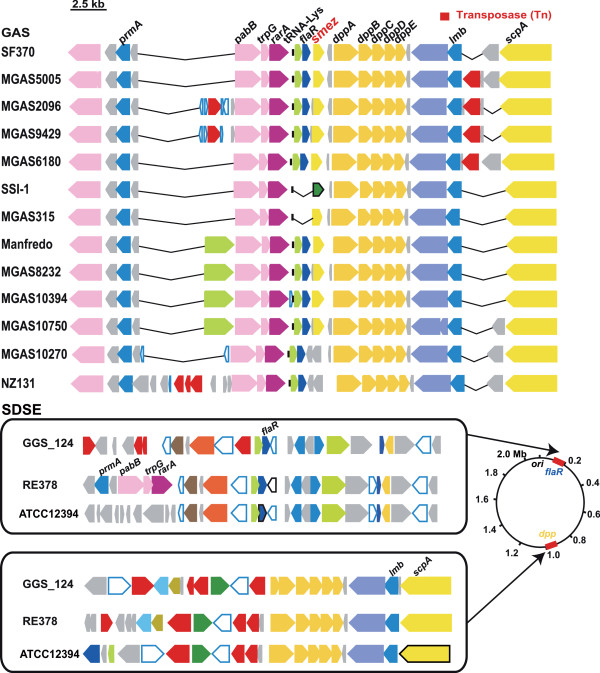
**Synteny mapping of the *smez* region among SDSE and GAS genomes.** Each position (bp) on each genome is shown in Additional file 10. The genes flanking *flaR* and the *dpp* operon were inversely aligned in three SDSE strains. The genomic locations of *flaR* and the *dpp* operon in SDSE and GAS are also shown.

The complete sequencing of the entire SDSE genome enabled us to gain insight into the origin of streptococcal SAgs. To elucidate the evolutionary pathways of streptococcal SAgs, it was first necessary to distinguish orthologous from paralogous SAgs in streptococcal genomes. This can be accomplished by syntenic mapping of the genes in regions of interest. Since *speG* and *smez* are conserved in almost all GAS genomes, but show low sequence similarities at the nucleotide level (35%), they are likely distinct direct descendants of ancestral streptococcal SAgs. Inasmuch as some of the regions surrounding *speG* in the GAS genomes harbor genes encoding putative transposases, which mediate the mobilization of the surrounding genes (Figures 1 and 2), and since *speG* and *smez* have low GC contents compared with their surrounding regions (data not shown), we cannot exclude the possibility that *speG* and *smez* are also paralogous genes. By analyzing the entire genome of the SDSE strain GGS_124 [DDBJ: AP010935] [4], we could compare its genome with those of other bacteria. We found that this SDSE genome was 65% identical in sequence to that of the GAS genome (Additional file 2), the highest to date among sequenced bacterial genomes. This finding strongly suggested that SDSE and GAS evolved from a common ancestor, despite SDSE harboring only the putative SAg gene *speG*. We therefore analyzed the syntenic homology of the *speG* regions of GGS_124 and GAS strains. We found that, at the amino acid level, *speG* in GGS_124 was 79% to 83% similar to the *speG* regions of GAS strains. 

To exclude the possibility that *speG* was acquired from a streptococcal phage, we compared the 50 kb sequences surrounding *speG,* a size sufficient to detect sequences derived from prophages. Synteny maps of the respective *speG* regions were essentially conserved in GAS strains and GGS_124 (Figure 1A), except for MGAS10750, which did not harbor the *speG* sequence present in the corresponding *speG* regions of GAS and GGS_124. We found that the *speG* region of each GAS genome contains two to ten genes, which encode factors similar to mobile elements and phage-related genes, such as transposase, IS and co-activator of prophage gene expression. In contrast, these mobile elements could not be detected in the corresponding *speG* region of GGS_124 (Figure 1A). The synteny of the regions surrounding the *speG* gene was highly conserved in eight GAS genomes (i.e. SF370, MGAS5005, MGAS2096, MGAS9429, MGAS10270, SSI-1, NZ131, and MGAS6180), each of which contains seven to eight transposase- and phage-related genes. These regions were 94% to 100% identical with each other. In the Manfredo genome, we found that IS*1239*, which is widely distributed in various isolates of GAS [22], had been inserted into the *speG* coding sequence, resulting in *speG* being a pseudogene in this strain (Figure 1A). In the MGAS8232 genome, IS*1239* flanked *speG*.

Although a previous study suggested that *speG* transferred from SDSE to GAS [23], our results clearly indicate that the synteny surrounding *speG* in the GAS and GGS_124 genomes has been essentially conserved and that modifications of this context, by insertion of mobile elements, occurred only in GAS strains. These results strongly suggest that *speG* in GAS and SDSE is an orthologous, not a xenologous, gene, the latter defined as a gene displaced by horizontal transfer from another lineage [24]. Moreover, *speG* in GAS and SDSE is a descendant of an ancestral streptococcal SAg and has been conserved in evolution.

**Figure 3:**
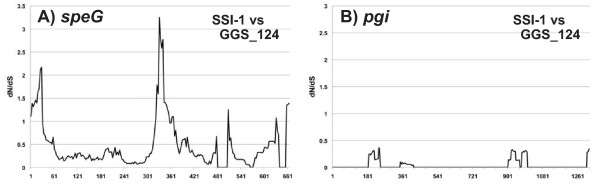
**Window analysis calculating dN/dS between GAS and SDSE.** dN/dS was calculated as the ratio of nonsynonymous (dN) to synonymous (dS) substitution rates of gene pairs in *speG* (**A**) and *pgi* (**B**) from GAS and SDSE genomes. The figure shows only the results of comparisons between SSI-1 (GAS) and GGS_124 (SDSE). The X-axis represents nucleotide position.

We next performed amino acid substitution analysis, based on window analysis, to estimate the number of non-synonymous (dN) and synonymous (dS) substitutions per site for *speG* and *pgi*, a housekeeping gene that encodes glucose-6-phosphate isomerase, in GAS and SDSE strains. The ratio of non-synonymous to synonymous substitution rate (dN/dS) can be used to determine patterns of molecular evolution, with dN/dS > 1 indicating positive selection, dN/dS = 1 indicating neutral selection, and dN/dS < 1 indicating purifying selection. A comparison of *speG* in GAS and SDSE genomes revealed five peaks with dN/dS > 1 (Figure 3A), suggesting positive selection in these five regions. Crystal structures of SpeC, the SAg protein structurally most similar to SpeG, suggested that several highly conserved domains, including Lys88-Leu97, Gln154-Thr167, and Asp188-Phe197, are important for protein function [25]. Interestingly, the dN/dS ratios in these conserved regions were low, suggesting that positive selection pressure in *speG* operates to conserve a function other than superantigenic activity, because none of the SDSE clinical strains expressed SpeG proteins [13-15]. For comparison, we performed window analysis on the *pgi* gene, but no dN/dS peak above one was observed (Figure 3B), despite this gene being adjacent to *speG*. 

### Comparison of the *speG* region among SDSE strains

Although the synteny of *speG* regions of GAS strains and GGS_124 has been highly conserved, about 50% of SDSE strains do not harbor *speG*[13-15]. We therefore selected nine SDSE isolates, five with (GGS_124, 163, 164, 168 and 170) and four without (RE378, SDSE_118, 160, and 165) *speG* (Additional file 1). Following direct genome sequencing (GGS_124 and RE378) or PCR amplification using *speG* specific primers (Additional file 3), we compared the sequences of these nine strains. Each of these isolates harbored a different *emm* type (Additional file 1), widely used to type GAS and SDSE strains [26]. We also included the full sequence of the ATCC 12394 genome [GenBank: CP002215], an SDSE that does not harbor *speG*[27].

When we analyzed the genetic structures surrounding *speG* (12 to 60 kb) in these SDSE isolates (Figure 1B), we found that, in general, these structures were highly conserved, especially in the 12 kb regions between *pgi* (pink) and *perR* (blue), but that *speG* itself and its corresponding regions were not. Outside these 12 kb regions, we found that most of these strains contained 1 or 2 coding sequences similar to transposase or IS elements, including several that appeared to be common to the sequenced GAS genomes.

Remarkably, all four *speG*-negative strains (e.g. RE378, SDSE_118, 160, and 165) showed the insertion of an approximately 20 kb fragment between the hypothetical protein gene (locus_tag: SDEG_1990) and the gene similar to peptidoglycan endo-beta-N-acetylglucosaminidase (locus_tag: SDEG_1992) present in the GGS_124 genome, replacing *speG* (locus_tag: SDEG_1991) at the exact same site. These 20 kb fragments were composed of 19 or 22 coding sequences, which were similar to genes derived from evolutionally distant species such as *Clostridium botulinum* and *C*. *tetani*. However, the arrangements of these genes did not exactly match those of the clostridial genomes (data not shown), with most coding sequences sharing <60% similarity (e.g. Additional file 4). In contrast, genetic structures other than these 20 kb fragments were highly conserved among the *speG*-positive and -negative strains (Figure 1B). These findings indicated that synteny had been conserved in the regions surrounding *speG*, or the inserted 20 kb fragments, of these SDSE strains.

### Analysis of the expression of *speG* region genes

**Figure 4:**
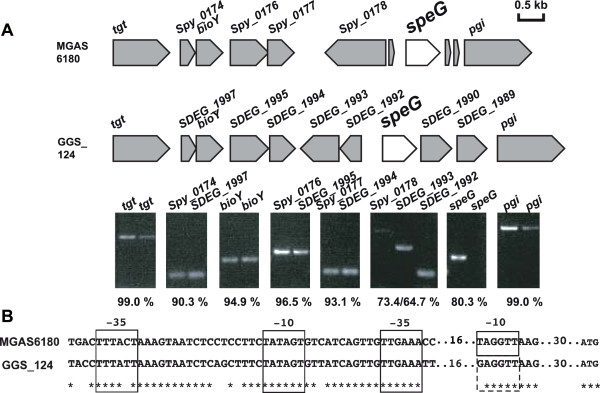
**Expression of genes surrounding *speG*.** (**A**) RT-PCR analysis of the expression of the eight or nine genes surrounding *speG* in GGS_124 (SDSE) and MGAS6180 (GAS). The amino acid identity of each gene (%) in these two strains is indicated below each gene. (**B**) Putative predicted promoter regions at -10 bp and -35 bp of *speG* in GGS_124 and MGAS6180. The mutation in the putative -10 bp region in GGS_124 made it no longer a putative promoter region; it is therefore enclosed in a dotted line box.

Our finding, that the genomic structures surrounding *speG* in GAS and SDSE strains are highly conserved, even in *speG*-negative SDSE strains, suggested that *speG* in some ancestral SDSE strain had been replaced by a 20 kb fragment soon after the speciation of GAS and SDSE. These types of replacement may take place more frequently among non-functional than among functional genes. To test this hypothesis, we analyzed the expression of genes surrounding *speG* by RT-PCR, using total RNA isolated from two strains, GGS_124 (SDSE) and MGAS6180 (GAS). In contrast to MGAS6180, which expressed all of the genes analyzed in this study (Figure 4A), GGS_124 expressed all except *speG*, a difference that may be due to differences in nucleotide sequences at the *speG* promoter site. When we looked for putative promoter sites −35 bp and −10 bp upstream of *speG*, we found two (Figure 4B), both of which displayed a single nucleotide difference between GGS_124 and MGAS6180. We also identified one mutation each in the second promoter candidate of GAS strains Manfredo, SF370, and MGAS5005, although these mutations did not affect the predicted promoter score (data not shown). In contrast, the mutations in the GGS_124 promoter candidates affected the promoter scores [28], making them no longer candidate promoters (data not shown). Our results were in good agreement with previous findings [25], in that none of the SDSE clinical strains expressed SpeG proteins, despite possessing intact *speG* genes. 

### Analysis of the *smez* region

We also analyzed the genome context surrounding *smez*, another chromosomally encoded SAg, in GAS and SDSE strains. We found that the synteny of approximately 20 kb regions containing *smez* (see details for MGAS10270 and NZ131, below) were highly conserved in all GAS strains sequenced (Figure 2). Of the 13 completely sequenced GAS strains, 11 harbored *smez* genes, primarily at approximately 1.7 Mb. Although *smez* was present at the same site in SSI-1 as in the other GAS genomes, the former was functionally inactive due to a frame-shift mutation. In contrast, the MGAS10270 and NZ131 genomes did not contain *smez* fragments, even at other locations, despite their corresponding surrounding genome structures being highly conserved when compared with the other GAS strains. All GAS genomes contained highly similar *dpp* operons (*dppA*, *dppB*, *dppC*, *dppD*, and *dppE*) immediately downstream of *smez*, and all contained *flaR* and *trpG*, located upstream of *smez* coding sequences (Additional file 5).

Analysis of the GGS_124, RE378, and ATCC 12394 strains revealed that none contained fragments similar to the 702 bp *smez* coding sequence derived from the SF370 genome. We therefore searched for *flaR* and the *dpp* operon, which were highly conserved in the *smez* flanking regions of GAS genomes (Figure 2). In these three SDSE genomes, *flaR* was located at about 0.2 Mb, whereas the *dpp* operon was located at about 0.9 Mb, far from the position of *flaR* (Figure 2 and Additional file 5). Furthermore, synteny of the regions surrounding *flaR* and the *dpp* operon was not well conserved in these three SDSE genomes, suggesting rearrangement of the genome context. The *flaR* gene and the *dpp* operon show high similarities in GAS and SDSE (Additional file 5), with concomitant sequences observed only in GAS and SDSE but not in other streptococci (data not shown).

We next plotted cumulative TA-skew diagrams of the three sequenced SDSE chromosomes (GGS_124, RE378, and ATCC 12394). Use of a similar method on 12 sequenced GAS genomes showed that all cumulative TA-skew curves of GAS genomes displayed a V-shape, interrupted by segments of steeper slopes, called steep-slope regions (SSRs) [29]. Diagram distortions including SSRs are thought to correspond to positions in which foreign genetic elements are integrated, including prophage-related genes [30], horizontally acquired elements [31], and pathogenicity islands [29], and in which genome rearrangements occur [29]. The SSR was conserved among GAS strains, with *smez* at the border of the SSR, suggesting that this region is predisposed to be unstable [29].

**Figure 5:**
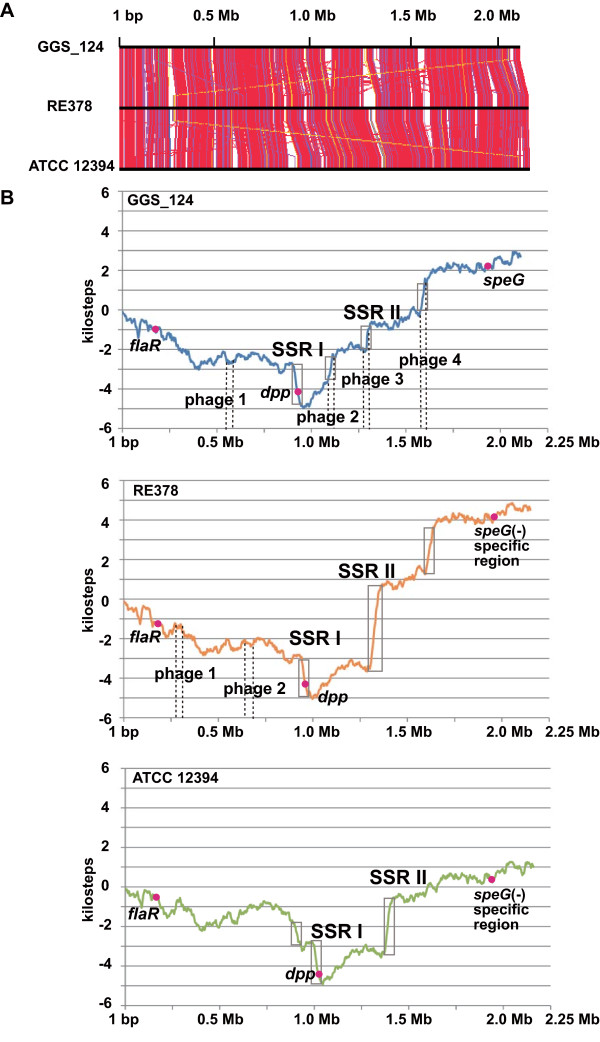
**Genome wide comparison among three SDSE genomes.** (**A**) Genome rearrangement map among the three SDSE genomes. Each line joins two orthologues and the color of the lines represents the percentage of similarity between orthologous gene products (blue ≤ 40% ≤ green ≤ 60% ≤ yellow ≤ 70% ≤ orange ≤ 80% ≤ magenta ≤ 90% ≤ red). (**B**) Cumulative TA-skews for the three SDSE genomes. Gray boxes represent SSRs. Each page region in the GGS_124 and RE378 genomes is indicated with broken lines. The X-axis represents nucleotide position.

Cumulative TA-skew curves of the three SDSE genomes formed a V-shape, similar to the GAS genomes (Figure 5B). GGS_124 contained four SSRs, whereas RE378 and ATCC 12394 contained three, some of which were located at similar positions (e.g. SSRs I and II, Figure 5B). These three SDSE genomes showed no evidence of massive genomic transversion or transition events (Figure 5A). Three of the four SSRs in the GGS_124 genome corresponded to prophages ΦGGS_124.1, ΦGGS_124.2, and ΦGGS_124.4, with the fourth being a newly identified prophage-like element. In the RE378 genome, there was no correlation between the localization of two prophage-like elements and SSRs. Since SSR I and SSR II are conserved in all three genomes (Figure 5B), we further analyzed the genome contexts of SSR I and SSRII. The SSR I sequences from the three SDSE genomes varied in size and number of CDSs, with sizes ranging from 44 to 62 kb. The number of CDSs included in SSR I also varied among the three SDSE genomes, but their core gene content was conserved, with high (> 95%) identity in the three genomes (Additional file 6). The *Dpp* operon, which is located in the region flanking *smez* in GAS strains (Figure 2), was contained in SSR I. This result is in good agreement with findings showing that the *dpp* operon was located in a non-phagic SSR and conserved in all sequenced GAS genomes [29]. SSR II from the three SDSE genomes also varied in size, from 23 to 64 kb, and number of CDSs. Although SSR II in the GGS_124 genome, at 1287230–1299098 bp, corresponded to the prophage region (Figure 5B), the core gene content in other non-phagic regions was conserved in all three genomes with high (> 95%) similarities (Additional file 6). The presence of these two non-phagic SSRs suggests that another rearrangement event was involved in the formation of these SSRs. In one of the events in SSR I, *smez* was lost from the SDSE genome because the *dpp* operon was a part of this SSR. These findings and the absence of *smez* from almost all SDSE strains [13] suggested that *smez* is a direct descendant of a common ancestor of streptococcal SAgs. Although this gene was conserved in GAS genomes, it was lost from SDSE due to a massive genome rearrangement that occurred after the speciation of SDSE and GAS. 

### Identification of conserved enterotoxin like and staphylococcal superantigen like genes in all *S*. *aureus* genomes

**Figure 6:**
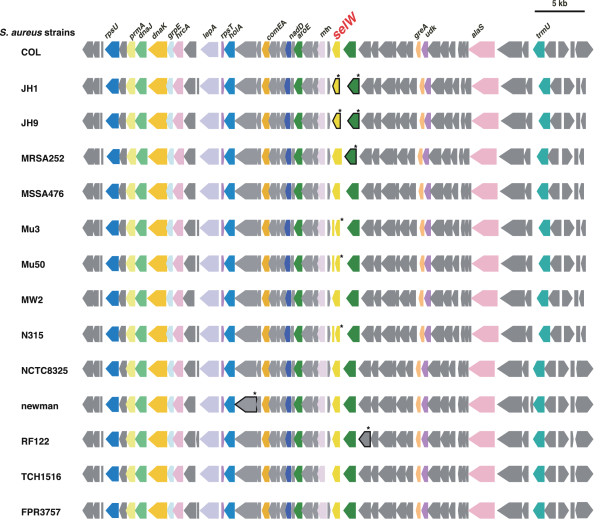
**Synteny mapping of the *selW* region among *S*. *aureus* strains.** Each position (bp) on each genome is shown in Additional file 10. Pseudogenes are marked with asterisks.

To determine the evolutionary pathway of staphylococcal SAgs, we analyzed all 14 *S*. *aureus* genomes to determine whether their core chromosomes harbor orthologous staphylococcal enterotoxin-like (SEl) gene(s). We observed an SE-like gene (locus_tag SA1430) in *S*. *aureus* N315; this gene was designated the staphylococcal enterotoxin-like W (*selW*) gene according to guidelines [21] (Figure 6). We found no other candidate orthologous SE gene in these core genomes (data not shown). Surprisingly, SElW has not yet been functionally analyzed, despite extensive study of staphylococcal SAgs. This may be due, at least in part, to *selW* being annotated as SEA in the *S*. *aureus* genomes. We found, however, that the amino acid sequence of SElW is only 36% identical to that of SEA, although phylogenetic analysis indicated that SEA is gene most similar to SElW. 

**Figure 7:**
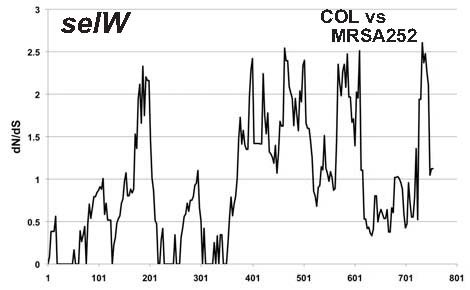
**Window analysis calculating dN/dS of *selW*.** dN/dS was calculated as the ratio of nonsynonymous (dN) to synonymous (dS) substitution rates of gene pairs in *selW* from two MRSA genomes. The figure shows only the comparison of COL and MRSA252. The X-axis represents nucleotide position.

We found that the *selW*s are located at about 1.6 to 1.7 Mb in the *S*. *aureus* genome, proximal to the *pfs* gene encoding 5'-methylthioadenosine nucleosidase/S-adenosylhomocysteine nucleosidase. Synteny of the *selW* locus, including the proximal 50 kb regions, was found to be conserved in all 14 *S*. *aureus* genomes available on the database (Figure 6). In contrast to other staphylococcal SAgs described to date, we detected no factor related to mobile genetic elements in these 50 kb regions. These findings strongly suggest that *selW* is a direct descendant of an ancestral staphylococcal SAg. To analyze whether *selW* has been subjected to positive selection, we performed window analysis for *selW*. Since no orthologous candidate genes were detected in other staphylococci or related bacteria, we performed our analysis using two *selW* genes from two different staphylococcus genomes. We found that several peaks had dN/dS ratios >1 (Figure 7), although these genes were derived from the same species. The ratios were especially high in the 3’ region of *selW*. These results suggested that *selW* has been subjected to positive selection.

**Figure 8:**
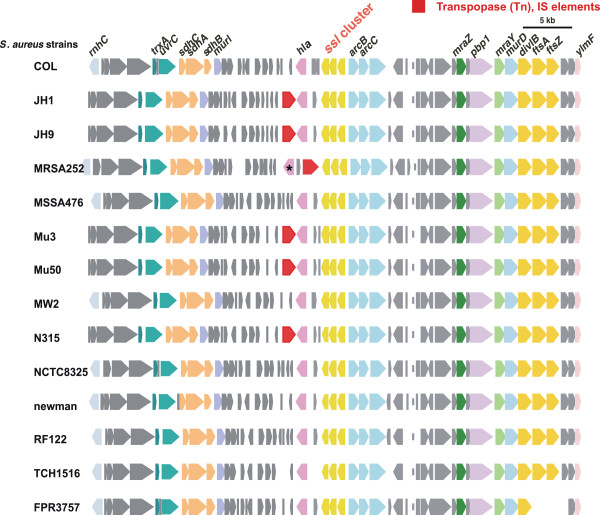
**Synteny mapping of the *ssl *cluster region among *S. aureus *strains.** Each position (bp) on each genome is shown in Additional file 10. Pseudogenes are marked with asterisks, and transposons and insertion sequences are shown in red.

**Figure 9:**
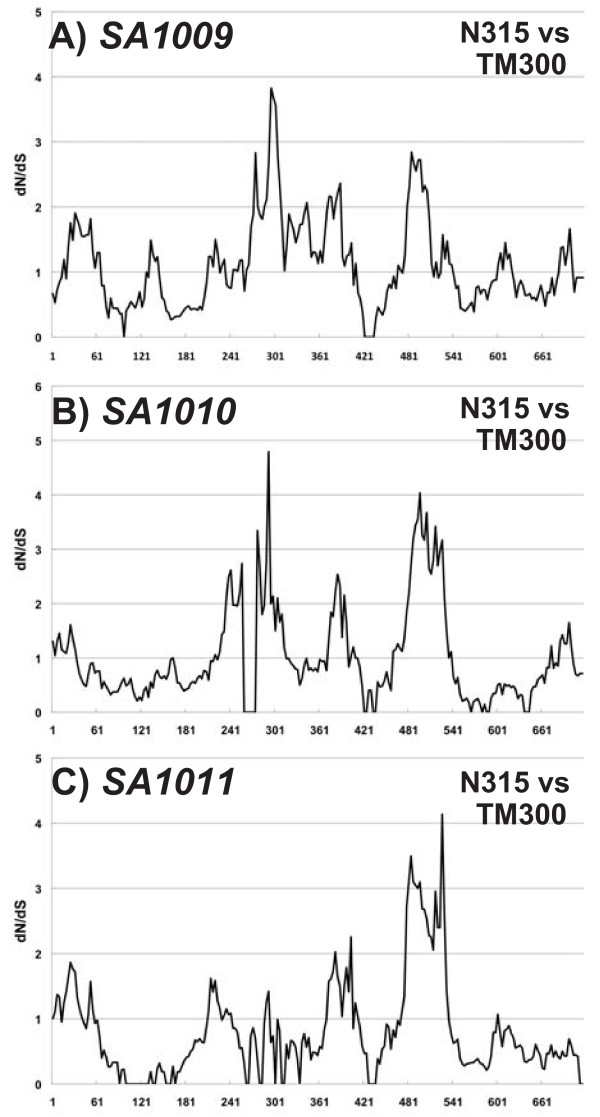
**Window analysis calculating dN/dS of *ssl*.** dN/dS was calculated as the ratio of nonsynonymous (dN) to synonymous (dS) substitution rates of gene pairs in *ssl-12* and Sca_0905 (**A**), *ssl-13* and Sca_0905 (**B**), and *ssl-14* and Sca_0905 (C) from *S. aureus* and *S. carnosus* subsp. *carnosus* genomes. The figure shows only the comparison between N315 (*S. aureus*) and TM300 (*S. carnosus* subsp. *carnosu*). The X-axis represents nucleotide position.

Analysis of the 14 *S*. *aureus* genomes revealed three highly conserved *ssl*-like gene(s) in the core chromosome (*ssl-12*, *ssl-13* and *ssl-14*; locus_tags SA1009, 1010, and 1011, respectively, in *S*. *aureus* N315) (Figure 8). Each of these genes showed approximately 30% amino acid identity to *ssl* gene products. No other candidate *ssl-*like genes were present in any of the *S*. *aureus* genomes (data not shown). These genes have often been regarded as exotoxin- or superantigen-like genes, but they have not yet been functionally analyzed. The product of the SA1011 gene (*S*. *aureus* N315 *ssl-14*) had a C-terminal β-grasp domain (Pfam02876), which is a structural signature of SAg, whereas none had an N-terminal oligosaccharide-binding domain. The three genes are located at positions 1.1 to 1.3 Mb of the *S*. *aureus* genome, upstream to the ornithine carbamoyltransferase (*arcB*) and downstream to the alpha-hemolysin (*hla*) gene. In some strains such as JH1 and MRS252, *hla* is replaced by transposons, but synteny of the upstream region remains highly conserved among these strains (Figure 8). Well-described SSLs are usually located in staphylococcal PIs, tandem structures consisting of three to ten SSL genes [16,18,32]. Since these *ssl*-like genes constitute a cluster of three homologous genes, their tandem structure in the PI is likely derived from their replication. To determine whether these *ssl*-like genes had been subjected to positive selection, we performed window analysis for *ssl*, using the three *ssl*-like genes, *ssl-12*, *ssl-13* and *ssl-14*, located in the core chromosome. For comparison, we used Sca_0905 (Sc-set) derived from *S. carnosus* subsp. *carnosus* TM300, because its product showed significant similarities with products of the *ssl*-like cluster [33]. For each *ssl*-like gene, we observed several dN/dS ratios greater than one (Figure 9). Although the three genes had slightly different patterns, high ratios were especially present in the middle and 3’ regions of *ssl*. These findings suggest that positive selection has operated on these regions to create variations in staphylococcus SAgs and strongly suggest that these *ssl*-like genes are direct descendants of an ancestral staphylococcal SSL (SET). 

*S. aureus* also harbors many types of SAgs, such as TSST-1, SEs, and SSLs. We identified a relatively unknown staphylococcal SAg, *selW*, and an *ssl* gene cluster, both of which are conserved in all *S*. *aureus* genomes examined to date. Moreover, we found that each of these genes was located in the same chromosomal region of the *S*. *aureus* genomes, not within any mobile elements. The highly syntenic conservation of *selW an*d the *ssl* gene cluster among *S*. *aureus* genomes and their similarity to SEs and SSLs, respectively, suggest that they are likely the direct descendants of common ancestral SEs and SSLs, respectively.

### Evolutionary analysis of streptococcal and staphylococcal SAgs and SSL

**Figure 10:**
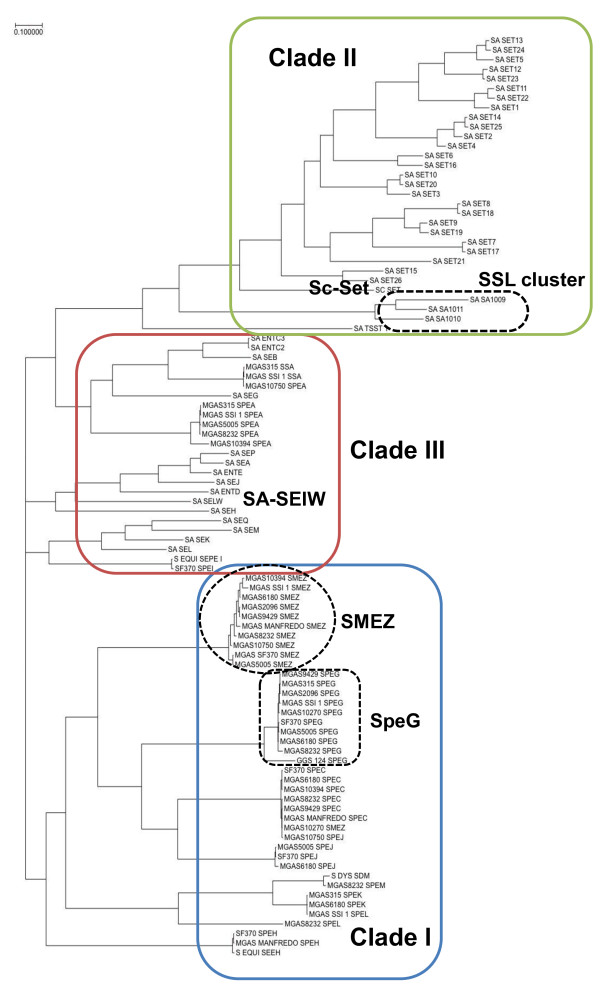
**Phylogenetic tree of streptococcal and staphylococcal SAgs and SSL nucleotide sequences.** The phylogenetic tree was constructed using the Bayesian MCMC method, with 100,000 generations. The resultant potential scale reduction factor was 1.078. Essentially the same result was obtained by changing the number of generations and by using the amino acid evolution model (data not shown). The nucleotide sequences used for alignment are shown in Additional file 9. The resulting phylogenetic tree was composed of three clades, with clade I including only streptococcal SAgs, clade II including only staphylococcal SSLs, and clade III including SAgs from both species. Orthologous gene products, including SpeG and SMEZ in clade I, SSL-like proteins in clade II and SElW in clade III, are emphasized.

To determine the entire evolutionary pathway of streptococcal and staphylococcal SAgs and SSL, we constructed an evolutionary tree based on their nucleotide and amino acid sequences, including the newly identified SElW and SSL-like cluster (Figure 10), using a Bayesian Markov chain Monte Carlo (MCMC) method. This approach, based on comparisons of the posterior probability of phylogenetic trees, allowed us to trace the evolutionary pathway of SAg in chronological order. In the resulting phylogenetic tree, streptococcal and staphylococcal SAgs and SSL could be divided into three groups, with clades I and II consisting of streptococcal SAgs and staphylococcal SSLs, respectively, and clade III consisting of both staphylococcal SEs and streptococcal SAgs (Figure 10). We found that each clade included at least one direct descendant in the core chromosome (e.g. SpeG and SMEZ for clade I, SSL-cluster and Sc-Set for clade II and SA-SElW for clade III). A phylogenetic tree based on amino acid sequences was similar to that of the tree based on nucleotide sequences (Additional file 7). Because these trees were obtained by posterior probability analysis, it is highly likely that the SAgs in each clade evolved from their ancestors, which have been conserved in the core genome; i.e., SpeG and SMEZ in clade I, SSL in clade II and SElW in clade III. Some streptococcal SAgs in clade III, including SpeA, SpeI, and SSA were more closely related to staphylococcal SAgs, including SEA, and SEB than to other streptococcal SAgs. Furthermore, although SElW is located in the core chromosome (Figure 6), all of the streptococcal SAgs belonging to clade III are located in their prophages. These results suggest that some ancestral genes of streptococcal SAgs descended from staphylococcal *selW* and that ancestral genes of *selW* were transferred from staphylococcal to streptococcal genomes.

The physiological activities and three-dimensional structures of SAgs are quite similar in streptococci and staphylococci. Although many studies have focused on staphylococcal SAgs in mobile elements, little is known about staphylococcal SAg-related gene(s) located on the core chromosome. To analyze the relationship among SAgs, we employed the Bayesian MCMC method. Although the phylogenetic tree we obtained was similar to that observed previously report [23], the method we used makes possible the determination of the temporal evolution of SAgs. Evolutionary analysis of the streptococcal and staphylococcal SAgs, and their related products, SSLs, showed that those molecules could be divided into three clades, each of which contains at least one direct descendant of an ancestor. SAgs of clades I and III consist of streptococcal and staphylococcal SAgs, respectively. In contrast, clade III is a mixture of streptococcal and staphylococcal SAgs, containing only SELW of *S. aureus*.

**Figure 11:**
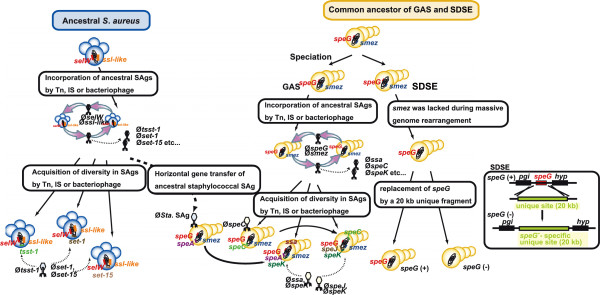
**Schematic diagram of the molecular evolution of streptococcal SAgs, SEs and SSLs.** This model was based on the results of this study. *S*. *aureus* and *Streptococcus* are shown in blue and orange, respectively, with the two lineages of SAgs consisting of those derived from these two species. The first step of SAg diversification consisted of the mobilization of ancestral SAgs (ancestral *selW* and *ssl*-like genes in *S*. *aureus*, and *speG* and *smez* in *Streptococcus*) by mobile elements. During their repeated transfer among bacteria, genes that might have been incorporated into mobile elements diversified, were transferred to *Streptococcus* and became ancestral to streptococcal SAgs, similar to *S*. *aureus* SAgs such as *speA* and *ssa*. In streptococcal lineages, the ancestral bacterium of GAS and SDSE harbored ancestral *speG* and *smez*. After speciation of GAS and SDSE, *smez* was lost from the SDSE lineage during massive genome rearrangements, whereas *speG* in SDSE was inherited from ancestral GAS and SDSE. In the GAS lineage, horizontal transfer of SAgs by streptococcal phages was linked to their diversification.

Based on these findings, we propose a model multi-step pathway for the evolution of SAgs (Figure 11). In step 1, the ancestors of streptococcal SAgs, SEs, and SSLs were acquired by ancestral bacteria. Detailed analyses of *speG*, *smez*, and *selW* were unable to determine additional ancestral forms, whereas the products of the *ssl*-like cluster were significantly similar to a product of Sca_0905 (Sc-set) derived from *S. carnosus* subsp. *carnosus* TM300 [33]. Although Sca_0905 itself is not conserved in *S*. *aureus* genomes, we found that the surrounding 50 kb regions were highly conserved in *S. carnosus* and *S*. *aureus* (Additional file 8)*.* Thus, the *ssl*-like cluster may have arisen by multiplication of the ancestor of the Sca_0905 gene present in ancestral *Staphylococcus.* This hypothesis may be clarified when more genome sequences of *S. carnosus* become available. In step 2 of the evolutionary pathway, ancestral *smez* was likely deleted from the ancestral SDSE during a massive genome rearrangement driven by the SSR [29], whereas the ancestral *speG* survived in ancestral GAS and SDSE. Since *speG* is a dormant gene, it was likely replaced by a 20 kb fragment in ancestral SDSE strains soon after the speciation of GAS and SDSE. In step 3, the ancestral SAgs and SSLs were incorporated into mobile genetic elements, most likely phages, by chance, and transferred among bacterial strains. Phages derived from GAS can infect other species of *Streptococcus*[34-36]. A recombination-based model has been proposed for streptococcal toxins, including SAg dissemination among prophages [37]. This type of recombination event may drive molecular diversity. Bacteriophages in *S*. *aureus* have wide host ranges and potent lytic capability, and some carry staphylococcal SAgs such as SEC and TSST-1 [38]. It is highly likely that SEs and SSLs were duplicated during the transfer among bacteria. In step 4 of the evolutionary pathway, horizontal gene transfer occurred across species barriers. SaPI1 containing TSST-1, one of the most frequently observed SAgs in *S*. *aureus*, was shown to have been transferred to an evolutionarily distant species, *Listeria monocytogenes*, by staphylococcal phages [39]. Since no SAgs in clade I were closely similar to staphylococcal SAgs, horizontal transfer of SAgs likely occurred exclusively from *Staphylococcus* to *Streptococcus*. 

## Conclusion

Streptococcal SAgs are one of the important virulence factors involved in life-threatening diseases such as streptococcal toxic shock syndrome (STSS) and scarlet fever. At present, a total of 11 SAgs have been identified by GAS genome sequencing, with most GAS isolates possessing several SAg genes in their genomes. Although the diversity of SAgs is thought to arise during horizontal transfer, their evolutionary pathway has not been determined. To better understand SAg evolution, we sequenced the entire genome of SDSE, the closest relative of GAS, which harbors *speG* as its only SAg gene. Genome-wide comparisons of GAS and SDSE provided evidence that *speG* is the direct descendant of a common ancestor of the streptococcal SAg. Furthermore, we also detected previously undescribed inter-species horizontal SAg gene transfer events among three pathogens, *S*. *pyogenes*, *S. dysgalactiae* subsp. *equisimili*s and *S. aureus*. This study is the first time to describe the origin and evolution of SAgs in pathogenic streptococci and staphylococci. These findings suggest that horizontal gene transfer is a more ubiquitous genetic exchange system than previously known, and that it sometimes crosses interspecies barriers.

## Methods

### Bacterial strains and media

All *S*. *dysgalactiae* subsp. *equisimilis* (SDSE) strains used in this study were isolated from patients with invasive infections in different hospitals throughout Japan (Additional file 1). Each SDSE isolate was cultured in 5% sheep blood agar or Brain Heart Infusion medium at 37°C under 5% CO_2_ as described [8].

### Preparation of genomic DNA and sequencing

*Streptococci* were lysed as described [8], and genomic DNA was purified using Wizard® Genomic DNA Purification Kits (Promega).

PCR reactions were performed in volumes of 50 μl containing TaKaRa ExTaq DNA polymerase (TaKaRa), with amplification on a GeneAmp PCR System 9700 (Applied Biosystems). Primer sets for direct sequencing were based on GGS_124 and RE378 genome sequence data, with each set designed to amplify 5 kbp PCR products with 500 bp overlapping regions. The PCR primer set for the *speG*-specific region has been described [25] (see also Additional file 3). PCR products were electrophoresed on 1.0% agarose gels and purified using QIAquick PCR Purification kits (QIAGEN). All DNA fragments were sequenced on an ABI3100 DNA sequencer with a redundancy of 4.

### RNA preparation

All isolates were grown overnight in 10 ml of Brain Heart Infusion medium at 37°C under 5% CO2 in 15 ml conical tubes. The cells were harvested, total RNA was purified using RNeasy Mini Kits (QIAGEN), and RNA concentrations were measured using a NanoDrop™ 1000 spectrophotometer (Thermo Scientific).

### RT-PCR analysis of target genes

Total RNA was reversed transcribed into cDNA using Superscript III reverse transcriptase kits (Invitrogen) and oligo dT primers. PCR amplifications were performed using the primer sequences in Additional file 3. The PCR products were electrophoresed on 1.0% agarose gels and detected by UV-fluorescence after ethidium bromide staining.

### Bioinformatics and evolutionary analyses

Homology searches and IS searches were performed using BLAST ( http://blast.ncbi.nlm.nih.gov/Blast.cgi) and IS finder ( http://www-is.biotoul.fr/is.html), respectively. Cumulative TA skew analysis was performed using GenSkew ( http://genskew.csb.univie.ac.at/). Both window size and stepsize sequence length were set at 1000 bp. Rates of evolution were estimated by a Window Analysis of dN and dS, using the online interface of WINA 0.34 [40], in a sliding window size of 60 bp (20 codons) at 3 bp intervals. A phylogenetic tree was constructed with CLUSTALW ( http://clustalw.ddbj.nig.ac.jp/top-j.html), MrBayes 3.1.2 ( http://mrbayes.csit.fsu.edu/index.php) and TreeView X ( http://darwin.zoology.gla.ac.uk/~rpage/treeviewx/index.html) software. Sequences were manually corrected using GENETYX-Mac (GENETYX Co.) and gene analysis was performed by in silico molecular cloning (in silico Biology Co.).

### Nucleotide and amino acid sequence accession number

The DNA sequences of the region surrounding *speG* (10–60 kb) in each strain have been deposited in the DDBJ under the accession numbers listed in Additional file 1. Accession numbers for SAgs used in this study are listed in Additional file 9.

## Competing interests

The authors declare no competing financial interests.

## Authors’ contributions

KO performed the direct sequencing analyses and RT-PCR assays. KO and TM-A performed the bioinformatic analyses. KO, YS, SY-M and TM-A performed GGS_124 and RE378 genome sequence analyses. SY-M and KU provided isolates for the analyses. KU, TK and TM-A. supervised the project. JY and TK coordinated the project. KO and TM-A prepared the manuscript. All authors read and approved the final manuscript.

## Supplementary Material

Additional file 1 ***Streptococcus* isolates used in this study.** Click here for file

Additional file 2 **Homology of *S. dysgalactiae *subsp. *equisimilis *GGS_124 with other bacteria at the genome level.** Click here for file

Additional file 3 List of oligonucleotide primers used in this study. Click here for file

Additional file 4 **Features of *speG***-**negative strain specific regions.** Click here for file

Additional file 5 **Summary of *flaR* and the *dpp* operon in each *Streptococci* strain.** Click here for file

Additional file 6 List of genes present in SSRI and SSR II. Click here for file

Additional file 7 **Phylogenetic tree of streptococcal and staphylococcal SAgs and SSL amino acid sequences.** The phylogenetic tree was constructed using the Bayesian MCMC method, with 100,000 generations and a mixed amino acid evolution model. The resultant potential scale reduction factor was 1.078. Essentially the same result was obtained by changing the number of generations and using the amino acid evolution model (data not shown). The nucleotide sequences used for alignment are shown in Additional file 9. The resulting phylogenetic tree was composed of three clades, with clade I including only streptococcal SAgs, clade II including only staphylococcal SSLs, and clade III including SAgs from both species. Orthologous gene products, including SpeG and SMEZ in clade I, SSL-like proteins in clade II and SElW in clade III, are emphasized. *Yersinia* SAgs (YPMA, YPMB, and YPMC) were also included as an outgroup. Click here for file

Additional file 8 **Synteny mapping of the *set***-**containing regions of *Staphylococcus carnosus* TM300 and *S.aureus* strains.** Each position (bp) on each genome is shown in Additional file 10. Click here for file

Additional file 9 List of Accession numbers for SAgs used in this study. Click here for file

Additional file 10 List of Accession numbers for SAgs used in this study. Click here for file
