# Supplementary material for: Evolutionary paths of streptococcal and staphylococcal superantigens
Source: BMC Genomics. 2012 Aug 17;13:404. doi: 10.1186/1471-2164-13-404 (PMC3538662; doi:10.1186/1471-2164-13-404)
Supplement: Additional file 1 — Streptococcus isolates used in this study. [file 1471-2164-13-404-S1.doc]

**Additional file 1. *Streptococcus* isolates used in this study.**

|  | strain | M type | prophage | No. of SAg | SAg genes harbored | | | | | | | accession no. |
| --- | --- | --- | --- | --- | --- | --- | --- | --- | --- | --- | --- | --- |
|  | SF370 | M1 | 4 | 7 | *speC* | *speG* | *speH* | *speI* | *speJ* | *speK** | *smez* | AE004092 |
|  | MGAS5005 | M1 | 3 | 4 | *speA* | *speG* | *speJ* | *smez* |  |  |  | CP000017 |
|  | MGAS10270 | M2 | 5 | 3 | *speC* | *speG* | *smez* |  |  |  |  | CP000260 |
|  | SSI-1 | M3 | 6 | 6 | *speA* | *speG* | *speK* | *speL* | *ssa* | *smez* |  | BA000034 |
|  | MGAS315 | M3 | 6 | 4 | *speA* | *speG* | *speK* | *ssa* | *smez** |  |  | AE014074 |
|  | MGAS10750 | M4 | 4 | 3 | *speA* | *speJ* | *smez* |  |  |  |  | CP000262 |
| GAS | Manfredo | M5 | 5 | 3 | *speC* | *speH* | *smez* |  |  |  |  | AM295007 |
|  | MGAS10394 | M6 | 8 | 6 | *speA* | *speC* | *speH* | *speI* | *speK** | *smez* |  | CP000003 |
|  | MGAS2096 | M12 | 2 | 3 | *speA* | *speG* | *smez* |  |  |  |  | CP000261 |
|  | MGAS9429 | M12 | 3 | 3 | *speC* | *speG* | *smez* |  |  |  |  | CP000259 |
|  | MGAS8232 | M18 | 5 | 6 | *speA* | *speC* | *speG* | *speL* | *speM* | *smez* |  | AE009949 |
|  | MGAS6180 | M28 | 4 | 5 | *speC* | *speG* | *speK* | *speJ* | *smez* |  |  | CP000056 |
|  | NZ131 | M49 | 3 | 2 | *speG* | *speH* |  |  |  |  |  | CP000829 |
|  |  |  |  |  |  |  |  |  |  | | | |
|  |  | group | subsp. | hemolysis type | origin | *emm* type | *spegg* | accession no. |  | | | |
|  | GGS-124 | G | *equisimilis* | β | human | *stg480.0* | *spegg4* | AP010935 |  | | | |
|  | RE378 | G | *equisimilis* | β | human | *stg6792* | *-* | AP011114 |  | | | |
|  | GGS-118 | G | *equisimilis* | β | human | *stg6792.3* | - | AB479807 |  | | | |
|  | 160 | G | *equisimilis* | β | human | *stg652* | - | AB479808 |  | | | |
| SDSE | 163 | G | *equisimilis* | β | human | *stg643* | *spegg4* | AB479809 |  | | | |
|  | 164 | G | *equisimilis* | β | human | *stg485.2* | *spegg4* | AB479810 |  | | | |
|  | 165 | G | *equisimilis* | β | human | *stg6.1* | - | AB479811 |  | | | |
|  | 168 | G | *equisimilis* | β | human | *stg480.0* | *spegg2* | AB479812 |  | | | |
|  | 170 | G | *equisimilis* | β | human | *stC36.0* | *spegg3* | AB479813 |  | | | |
|  | ATCC 12394 | G | *equisimilis* | β | human | *stG166b* | *-* | CP002215 |  | | | |

* partial product
