# Supplementary material for: Evolutionary paths of streptococcal and staphylococcal superantigens
Source: BMC Genomics. 2012 Aug 17;13:404. doi: 10.1186/1471-2164-13-404 (PMC3538662; doi:10.1186/1471-2164-13-404)
Supplement: Additional file 2 — Homology of S. dysgalactiae subsp. equisimilis GGS_124 with other bacteria at the genome level. [file 1471-2164-13-404-S2.doc]

| Bacterial genome | Total score | Query coverage |
| --- | --- | --- |
| *Streptococcus pyogenes* MGAS10750 | 2.00E+06 | 66% |
| *Streptococcus pyogenes* MGAS5005 | 1.90E+06 | 65% |
| *Streptococcus pyogenes* MGAS10270 | 1.89E+06 | 65% |
| *Streptococcus pyogenes* SSI-1 | 1.86E+06 | 64% |
| *Streptococcus pyogenes* MGAS315 | 1.89E+06 | 64% |
| *Streptococcus pyogenes* SF370 | 1.88E+06 | 64% |
| *Streptococcus pyogenes* Manfredo | 1.99E+06 | 64% |
| *Streptococcus pyogenes* MGAS10394 | 2.03E+06 | 64% |
| *Streptococcus pyogenes* MGAS6180 | 1.86E+06 | 64% |
| *Streptococcus pyogenes* MGAS9429 | 1.86E+06 | 63% |
| *Streptococcus pyogenes* MGAS2096 | 1.87E+06 | 63% |
| *Streptococcus agalactiae* A909 | 5.27E+05 | 16% |
| *Streptococcus thermophilus* LMG18311 | 3.40E+05 | 10% |
| *Streptococcus thermophilus* CNRZ1066 | 3.41E+05 | 10% |
| *Streptococcus thermophilus* LMD-9 | 3.38E+05 | 10% |
| *Streptococcus pneumoniae* TIGR4 | 2.47E+05 | 8% |
| *Streptococcus pneumoniae* CGSP14 | 2.47E+05 | 8% |
| *Streptococcus pneumoniae* Hungary19A-6 | 2.42E+05 | 8% |

**Additional file 2. Homology of *S*. *dysgalactiae* subsp. *equisimilis* GGS_124 with other bacteria at the genome level.**
