# Supplementary material for: Evolutionary paths of streptococcal and staphylococcal superantigens
Source: BMC Genomics. 2012 Aug 17;13:404. doi: 10.1186/1471-2164-13-404 (PMC3538662; doi:10.1186/1471-2164-13-404)
Supplement: Additional file 3 — List of oligonucleotide primers used in this study. [file 1471-2164-13-404-S3.doc]

| Primer name | Sequense (5'-3') | Use |
| --- | --- | --- |
| GGS-speG-F | GCTATGGAAGTCAATTAGCTTATGCAG | *speG* typing |
| GGS-speG-R | GGCTCCCCGATGTATAACGCGATTCCG | *speG* typing |
| tgt-F | GGAACTCAGGCTACTGTTAAAACACAG | RT-PCR |
| tgt-R | GTTATCGTCCATAATAGCCTGACGTAC | RT-PCR |
| Spy0174-F | CGGACTGTTTTGGAATTGACCTTGACC | RT-PCR |
| Spy0174-R | GGATTAAAGGGACTTTGACAATAAGGAC | RT-PCR |
| Spy0175-F | GAACTTGTTAAGGTGGCTATGATGACC | RT-PCR |
| Spy0175-R | GGTATCTCCTGGAATAAAGACCAAGTT | RT-PCR |
| Spy0176-F | GACGGCTTTCAGTTATTGATGGATGCT | RT-PCR |
| Spy0176-R | ATTGTCAGGATCAATACCTTCTGGAGG | RT-PCR |
| Spy0177-F | CAGAAAGCTGAAATCCCCATTGGCTGTG | RT-PCR |
| Spy0177-R | CTGTCAAAATCTGGTAAAGGCTGTCTG | RT-PCR |
| Spy0178-F | GACAGTTGGTATGTCTGCGTGTCACAAC | RT-PCR |
| Spy0178-R | CTGGTAAGAGTCCATAGGTAAAGCCCG | RT-PCR |
| GGS-124-31814-F | GAGCGAGTCAAGTACTTCTATGAGGTG | RT-PCR |
| GGS-124-31814-R | CATAAGAGAAGCCACTATAATTGTCCC | RT-PCR |
| GGS-124-32599-F | CTGTGATGCTTGGCTTATCTGCTTG | RT-PCR |
| GGS-124-32599-R | CGGTAATCCGCCGAAGTAATCCAG | RT-PCR |
| Spy0180-speG-F | GCTATGGAAGTCAATTAGCTTATGCAG | RT-PCR |
| Spy0180-speG-R | GGCTCCCCGATGTATAACGCGATTCCG | RT-PCR |
| Spy0183-F | GTCCTCGAGTCATTTGCGGGACAAC | RT-PCR |
| Spy0183-R | GCTTCAACACCTGGTTGATCAAATGGG | RT-PCR |

**Additional file 3. List of oligonucleotide primers used in this study.**
