# Supplementary material for: Evolutionary paths of streptococcal and staphylococcal superantigens
Source: BMC Genomics. 2012 Aug 17;13:404. doi: 10.1186/1471-2164-13-404 (PMC3538662; doi:10.1186/1471-2164-13-404)
Supplement: Additional file 4 — Features of speG-negative strain specific regions. [file 1471-2164-13-404-S4.doc]

**Additional file 4. Features of *speG*-negative strain specific regions.**

| **Strain** | **Feature** | **Location** | **Similarity at amino acid level (%)** | **Product** | **Organism** | **Reference sequence** |
| --- | --- | --- | --- | --- | --- | --- |
| GGS_118 | CDS | complement(10990..14598) | 61.0 | putative pyruvate ferredoxin/flavodoxin oxidoreductase family protein | *Enterococcus faecalis* V583 | NP_815820.1 |
|  |  | complement(15612..16307) | 57.1 | propanediol utilization protein | *Clostridium botulinum* B str. Eklund 17B | YP_001885660.1 |
|  |  | complement(16317..17975) | 66.9 | acetaldehyde dehydrogenase (acetylating) | *Clostridium botulinum* E3 str. Alaska E43 | YP_001920780.1 |
|  |  | complement(17999..18604) | 48.6 | truncated carbon dioxide concentrating mechanism protein CcmK-like protein | *Clostridium tetani* E88 | NP_782063.1 |
|  |  | complement(18620..19045) | 60.1 | truncated ethanolamine utilization protein EutQ-likeprotein | *Clostridium botulinum* A3 str. Loch Maree | YP_001787500.1 |
|  |  | complement(19268..19564) | 68.7 | putative ethanolamine utilization protein EutN/carboxysome structural protein Ccml | *Alkaliphilus oremlandii* OhILAs | YP_001513920.1 |
|  |  | complement(19576..20418) | 67.8 | putative ethanolamine utilization protein EutJ family protein | *Clostridium botulinum* F str. Langeland | YP_001391407.1 |
|  |  | complement(20405..21037) | 30.1 | hypothetical protein | *Clostridium botulinum* E3 str. Alaska E43 | YP_001920772.1 |
|  |  | complement(21121..22251) | 50.0 | iron-containing alcohol dehydrogenase | *Clostridium phytofermentans* ISDg | YP_001558535.1 |
|  |  | complement(22248..22694) | 39.2 | putative EutP/PduV family GTP-binding protein | *Clostridium phytofermentans* ISDg | YP_001558534.1 |
|  |  | complement(22691..23038) | 62.8 | putative ethanolamine utilization protein EutS | *Clostridium tetani* E88 | NP_782068.1 |
|  |  | complement(23051..24040) | 58.9 | putative glycyl-radical activating family protein | *Clostridium phytofermentans* ISDg | YP_001558532.1 |
|  |  | complement(24102..26648) | 74.0 | formate C-acetyltransferase | *Clostridium phytofermentans* ISDg | YP_001558531.1 |
|  |  | complement(26709..28064) | 34.7 | putative aldehyde-alcohol dehydrogenase 2 | *Clostridium botulinum* B str. Eklund 17B | YP_001885646.1 |
|  |  | complement(28080..28388) | 68.6 | putative microcompartments protein | *Clostridium phytofermentans* ISDg | YP_001558529.1 |
|  |  | complement(28400..28699) | 79.6 | microcompartments family protein | *Clostridium botulinum* F str. Langeland | YP_001391415.1 |
|  |  | complement(29002..30009) | 50.8 | putative membrane spanning protein | *Clostridium phytofermentans* ISDg | YP_001558526.1 |
|  |  | complement(30043..30330) | 84.4 | bacterial microcompartments family protein | *Clostridium botulinum* E3 str. Alaska E43 | YP_001920782.1 |
|  |  | complement(30822..31697) | 41.5 | putative transcriptional regulator | *Clostridium phytofermentans* ISDg | YP_001558524.1 |
| 160 |  | complement(4430..8038) | 61.1 | putative pyruvate ferredoxin/flavodoxin oxidoreductase family protein | *Enterococcus faecalis* V583 | NP_815820.1 |
|  |  | complement(9053..9748) | 57.6 | propanediol utilization protein | *Clostridium botulinum* B str. Eklund 17B | YP_001885660.1 |
|  |  | complement(9758..11416) | 66.9 | acetaldehyde dehydrogenase (acetylating) | *Clostridium botulinum* E3 str. Alaska E43 | YP_001920780.1 |
|  |  | complement(11440..12045) | 48.6 | truncated carbon dioxide concentrating mechanism protein CcmK-like protein | *Clostridium tetani* E88 | NP_782063.1 |
|  |  | complement(12061..12693) | 50.0 | ethanolamine utilisation EutQ family protein | *Clostridium phytofermentans* ISDg | YP_001558540.1 |
|  |  | complement(12709..13005) | 68.7 | putative ethanolamine utilization protein EutN/carboxysome structural protein Ccml | *Alkaliphilus oremlandii* OhILAs | YP_001513920.1 |
|  |  | complement(13017..13859) | 67.8 | putative ethanolamine utilization protein EutJ family protein | *Clostridium botulinum* F str. Langeland | YP_001391407.1 |
|  |  | complement(13846..14478) | 30.1 | hypothetical protein | *Clostridium botulinum* E3 str. Alaska E43 | YP_001920772.1 |
|  |  | complement(14562..15692) | 50.0 | iron-containing alcohol dehydrogenase | *Clostridium phytofermentans* ISDg | YP_001558535.1 |
|  |  | complement(15689..16135) | 39.2 | putative EutP/PduV family GTP-binding protein | *Clostridium phytofermentans* ISDg | YP_001558534.1 |
|  |  | complement(16132..16479) | 62.8 | putative ethanolamine utilization protein EutS | *Clostridium tetani* E88 | NP_782068.1 |
|  |  | complement(16492..17481) | 58.6 | putative glycyl-radical activating family protein | *Clostridium phytofermentans* ISDg | YP_001558532.1 |
|  |  | complement(17543..20095) | 73.9 | formate C-acetyltransferase | *Clostridium phytofermentans* ISDg | YP_001558531.1 |
|  |  | complement(20150..21505) | 34.5 | putative aldehyde-alcohol dehydrogenase 2 | *Clostridium botulinum* B str. Eklund 17B | YP_001885646.1 |
|  |  | complement(21521..21829) | 68.6 | putative microcompartments protein | *Clostridium phytofermentans* ISDg | YP_001558529.1 |
|  |  | complement(21841..22140) | 79.6 | microcompartments family protein | *Clostridium botulinum* F str. Langeland | YP_001391415.1 |
|  |  | complement(22443..23450) | 50.8 | putative membrane spanning protein | *Clostridium phytofermentans* ISDg | YP_001558526.1 |
|  |  | complement(23484..23771) | 84.4 | bacterial microcompartments family protein | *Clostridium botulinum* E3 str. Alaska E43 | YP_001920782.1 |
|  |  | complement(24282..25157) | 41.8 | putative transcriptional regulator | *Clostridium phytofermentans* ISDg | YP_001558524.1 |
| 165 |  | complement(4453..8100) | 61.0 | putative pyruvate ferredoxin/flavodoxin oxidoreductase family protein | *Enterococcus faecalis* V583 | NP_815820.1 |
|  |  | complement(9075..9770) | 57.1 | propanediol utilization protein | *Clostridium botulinum* B str. Eklund 17B | YP_001885660.1 |
|  |  | complement(9780..11438) | 66.9 | acetaldehyde dehydrogenase (acetylating) | *Clostridium botulinum* E3 str. Alaska E43 | YP_001920780.1 |
|  |  | complement(11462..12067) | 48.6 | truncated carbon dioxide concentrating mechanism protein CcmK-like protein | *Clostridium tetani* E88 | NP_782063.1 |
|  |  | complement(12083..12715) | 60.1 | truncated ethanolamine utilisation EutQ family protein | *Clostridium botulinum* A3 str. Loch Maree | YP_001558540.1 |
|  |  | complement(12731..13027) | 68.7 | putative ethanolamine utilization protein EutN/carboxysome structural protein Ccml | *Alkaliphilus oremlandii* OhILAs | YP_001513920.1 |
|  |  | complement(13039..13881) | 67.8 | putative ethanolamine utilization protein EutJ family protein | *Clostridium botulinum* F str. Langeland | YP_001391407.1 |
|  |  | complement(13868..14527) | 30.1 | hypothetical protein | *Clostridium botulinum* E3 str. Alaska E43 | YP_001920772.1 |
|  |  | complement(14584..15714) | 49.7 | iron-containing alcohol dehydrogenase | *Clostridium phytofermentans* ISDg | YP_001558535.1 |
|  |  | complement(15711..16157) | 39.2 | putative EutP/PduV family GTP-binding protein | *Clostridium phytofermentans* ISDg | YP_001558534.1 |
|  |  | complement(16154..16501) | 62.8 | putative ethanolamine utilization protein EutS | *Clostridium tetani* E88 | NP_782068.1 |
|  |  | complement(16514..17503) | 58.6 | putative glycyl-radical activating family protein | *Clostridium phytofermentans* ISDg | YP_001558532.1 |
|  |  | complement(17565..20117) | 74.0 | formate C-acetyltransferase | *Clostridium phytofermentans* ISDg | YP_001558531.1 |
|  |  | complement(20172..21527) | 34.7 | putative aldehyde-alcohol dehydrogenase 2 | *Clostridium botulinum* B str. Eklund 17B | YP_001885646.1 |
|  |  | complement(21543..21851) | 68.6 | putative microcompartments protein | *Clostridium phytofermentans* ISDg | YP_001558529.1 |
|  |  | complement(21863..22162) | 79.6 | microcompartments family protein | *Clostridium botulinum* F str. Langeland | YP_001391415.1 |
|  |  | complement(22465..23472) | 50.8 | putative membrane spanning protein | *Clostridium phytofermentans* ISDg | YP_001558526.1 |
|  |  | complement(23506..23832) | 84.4 | bacterial microcompartments family protein | *Clostridium botulinum* E3 str. Alaska E43 | YP_001920782.1 |
|  |  | complement(24305..25180) | 41.5 | putative transcriptional regulator | *Clostridium phytofermentans* ISDg | YP_001558524.1 |
