# Supplementary material for: Evolutionary paths of streptococcal and staphylococcal superantigens
Source: BMC Genomics. 2012 Aug 17;13:404. doi: 10.1186/1471-2164-13-404 (PMC3538662; doi:10.1186/1471-2164-13-404)
Supplement: Additional file 5 — Summary of flaR and the dpp operon in each Streptococci strain. [file 1471-2164-13-404-S5.doc]

**Additional file 5. Summary of the *flaR* and *dpp* operon in each *Streptococci* strains.**

| Species | strain | *flaR* | | | | *dpp* operon | | | | |
| --- | --- | --- | --- | --- | --- | --- | --- | --- | --- | --- |
|  |  | location | strand | similarity (aa level) | comments | location | strand | similarity  (aa level) | | comments |
| *Streptococcus dysgalactiae* subsp. *equisimilis* | GGS_124 | 169256-169762 | - | 100% |  | 925836-930802 | - | 100% each | |  |
|  | RE378 | 184916-185476 | - | 95% |  | 959112-964085 | - | > 95% each |  | |
|  | ATCC 12394 | 171315-171809 | - | - | pseudogene | 1017818-1022785 | - | > 95% each |  | |
| *S. pyogenes* | MGAS8232 | 1724441-1724947 | + | 96% |  | 1726627-1731588 | + | > 95% each |  | |
|  | MGAS10394 | 1714355-1714861 | + | 95% |  | 1716534-1721501 | + | > 95% each |  | |
|  | MGAS10750 | 1749259-1749765 | + | 97% |  | 1751437-1756404 | + | > 95% each |  | |
|  | Manfredo | 1657725-1658231 | + | 97% |  | 1659910-1664877 | + | > 95% each |  | |
|  | MGAS10270 | 1720132-1720638 | + | 95% |  | 1722640-1727607 | + | > 95% each |  | |
|  | MGAS315 | - |  |  |  | 1733244-1738205 |  | > 95% each |  | |
|  | MGAS5005 | 1661525-1662031 | + | 96% |  | 1663710-1668671 | + | > 95% each |  | |
|  | MGAS9429 | 1657573-1658043 | + | 96% |  | 1659753-1664720 | + | > 95% each |  | |
|  | MGAS2096 | 1681458-1681928 | + | 96% |  | 1683638-1688605 | + | > 95% each |  | |
|  | SF370 | 1664431-1664937 | + | 96% |  | 1666616-1671577 | + | > 95% each |  | |
|  | SSI-1 | - |  |  |  | 1727024-1731991 | + | > 95% each |  | |
|  | MGAS6180 | 1691868-1692374 | + | 98% |  | 1694048-1699015 | + | > 95% each |  | |
|  | NZ131 | 1635460-1635966 | + | 97% |  | 1638159-1642982 | + | > 95% each |  | |
| *S. equi* subsp. *equi* | 4047 | 140865-141380 | - | 70% |  | - |  |  | | low similarities |
| *S. equi* subsp. *zooepidemicus* | MGCS10565 | 159590-160105 | - | 70% |  | - |  |  | | low similarities |
|  | H70 | 145770-146285 | - | 70% |  | - |  |  | | low similarities |
| *S. agalactiae* | A909 | 156538-157077 | - | 56% |  | 1908034-1912995 | - | > 90% each |  | |
|  | 2603 V/R | 141720-142259 | - | 56% |  | 1951337-1956298 | - | > 90% each |  | |
|  | NEM316 | 146252-146791 | - | 56% |  | 2001704-2006665 | - | > 90% each |  | |
| *S. uberis* | 0140J | - |  |  | low similarities ^*^ | - |  |  | | low similarities |
| *S. thermophilus* | CNRZ1066 | 78049-78564 | + | 69% |  | - |  |  | | low similarities |
|  | LMD-9 | 79295-79810 | + | 69% |  | - |  |  | | low similarities |
|  | LMG18311 | 80545-80991 | + | 69% | truncated | - |  |  | | low similarities |
| *S. mutans* | NN2025 | - |  |  | low similarities | - |  |  | | low similarities |
|  | UA159 | - |  |  | low similarities | - |  |  | | low similarities |
| *S. pneumoniae* | 70585 | 1385414-1385938 | + | 51% |  | - |  |  | | low similarities |
|  | ATCC 700669 | 1481193-1481717 | + | 49% |  | - |  |  | | low similarities |
|  | CGSP14 | 1439940-1440464 | + | 48% |  | - |  |  | | low similarities |
|  | Hungary19-A6 | 1381539-1382063 | + | 51% |  | - |  |  | | low similarities |
|  | TIGR4 | 1374510-1375034 | + | 49% |  | - |  |  | | low similarities |
|  | JJA | 1377194-1377718 | + | 48% |  | - |  |  | | low similarities |
|  | P1031 | 1344023-1344547 | + | 48% |  | - |  |  | | low similarities |
|  | D39 | 1300823-1301347 | + | 48% |  | - |  |  | | low similarities |
|  | R6 | 1300854-1301378 | + | 48% |  | - |  |  | | low similarities |
|  | Taiwan19F-14 | 807298-807822 | - | 49% |  | - |  |  | | low similarities |
|  | G54 | 1342592-1343116 | + | 49% |  | - |  |  | | low similarities |
| *S. gordonii* | Challis substr. CH1 | - |  |  | low similarities | - |  |  | | low similarities |
| *S.suis* | BM407 | - |  |  | low similarities | - |  |  | | low similarities |
|  | P1/7 | - |  |  | low similarities | - |  |  | | low similarities |
|  | SC84 | - |  |  | low similarities | - |  |  | | low similarities |
|  | 98HAH33 | - |  |  | low similarities | - |  |  | | low similarities |
|  | 05ZYH33 | - |  |  | low similarities | - |  |  | | low similarities |
| *S. sanguinis* | SK36 | 1340091-1340612 | + | 49% |  | - |  |  | | low similarities |

* low similarities means < 30% similarities at the amino-acids level.
