# Supplementary material for: Evolutionary paths of streptococcal and staphylococcal superantigens
Source: BMC Genomics. 2012 Aug 17;13:404. doi: 10.1186/1471-2164-13-404 (PMC3538662; doi:10.1186/1471-2164-13-404)
Supplement: Additional file 6 — List of genes present in SSRI and SSR II. [file 1471-2164-13-404-S6.pdf]

Additional file 6. List of genes shown in SSRI and SSR II.

	GGS_124						RE378		ATCC12394	
	locus_tag	Feature	5'	3'	Strand	gene	locus_tag	identity*	locus_tag	identity*
SSR I	SDEG_0914	CDS	902819	903232	+		GGS_0926	100	SDE12394_05150	100
	SDEG_0915	CDS	903383	903559	+	*lmb*	GGS_0928	99.4	SDE12394_05155	99.4
	SDEG_0916	CDS	903564	903932	+	*lmb*	GGS_0928	100	misc_feature	99.8
	SDEG_0917	CDS	903818	904318	+	*lmb*	GGS_0928	99.4	misc_feature	
	SDEG_0918	CDS	904315	904773	+	*htpA*	GGS_0929	100	SDE12394_05170	100
	SDEG_0919	CDS	905046	906005	+		GGS_0930	99.58	SDE12394_05175	99.69
	SDEG_0920	CDS	906329	906478	-		no CDS		no CDS	
	SDEG_0921	CDS	906471	907232	-		GGS_0931	98.17	SDE12394_05180	98.16
	SDEG_0922	CDS	907286	907576	-		GGS_0932	98.97	SDE12394_05185	98.28
	SDEG_0923	CDS	907849	908133	+		GGS_0933	99.65	SDE12394_05190	100
	SDEG_0924	CDS	908548	909810	+		GGS_0934	99.84	misc_feature	99.76
	SDEG_0925	CDS	909797	910471	+		GGS_0935	100	SDE12394_05205	99.41
	SDEG_0926	CDS	910484	911701	+		GGS_0936	98.85	SDE12394_05210	99.1
	SDEG_0927	CDS	911795	912448	+	*irr*	GGS_0937	99.39	SDE12394_05215	99.24
	SDEG_0928	CDS	912445	913833	+	*ihk*	GGS_0938	99.78	misc_feature	99.78
	SDEG_0929	CDS	913935	915563	+	*isp*	GGS_0939	99.39	SDE12394_05230	99.32
	SDEG_0930	CDS	915641	915892	+		GGS_0940	99.21	SDE12394_05235	98.41
	SDEG_0931	CDS	916224	916913	+		GGS_0941/_0942	99.71	SDE12394_05240/_5245	99.71
	SDEG_0932	CDS	917183	917860	+	*sic1-01*	GGS_0943	98.71	SDE12394_05250	99.38
	SDEG_0933	CDS	918282	921734	+	*scpB*	GGS_0944	98.29	misc_feature	99.42
	SDEG_0934	CDS	921779	921895	+		no CDS		no CDS	
	SDEG_0935	CDS	921986	922906	+	*lmb*	GGS_0945	99.46	SDE12394_05265	98.59
	SDEG_0936	CDS	922919	925387	+		GGS_0946	99.35	SDE12394_05270	99.43
	SDEG_0937	CDS	925455	925685	-		no CDS		no CDS	
	SDEG_0938	CDS	925555	925755	+		GGS_0947	100	SDE12394_05275	100
	SDEG_0939	CDS	925836	926462	-	*dppE*	GGS_0948	99.68	SDE12394_05280	100
	SDEG_0940	CDS	926446	927249	-	*dppD*	GGS_0949	99.13	SDE12394_05285	99.88
	SDEG_0941	CDS	927261	928082	-	*dppC*	GGS_0950	98.54	SDE12394_05290	99.03
	SDEG_0942	CDS	928079	929056	-	*dppB*	GGS_0951	97.85	SDE12394_05295	98.77
	SDEG_0943	CDS	929169	930224	-	*dppA*	GGS_0952	99.82	SDE12394_05300	99.79
	SDEG_0944	CDS	930218	930802	-	*dppA*	GGS_0952		SDE12394_05300	
	SDEG_0945	CDS	931273	931398	+		no CDS		no CDS	
	SDEG_0946	CDS	931426	932295	+		GGS_0955	99.08	SDE12394_05315	99.2

	SDEG_0947	CDS	932410	933633	+		GGS_0956	99.84	SDE12394_05320	99.75
	SDEG 0948	CDS	933734	934942	-		GGS 0957	99.5	SDE12394 05325	99.67
	SDEG 0949	CDS	935209	936186	+	*tra*	GGS 0958	94.01	SDE12394 05330	94.25
	SDEG 0950	CDS	936190	936291	+		GGS 0958		SDE12394 05330	
	SDEG 0951	CDS	936236	936652	+		GGS 0958		SDE12394 05330	
	GGS_124						RE378		ATCC12394	
	locus tag	Feature	5'	3'	Strand	gene	locus tag	identity*	locus tag	identity*
SSR II	SDEG 1350	CDS	1299629	1300990	-		GGS 1323	99.71	SDE12394 07245	99.62
	SDEG 1351	CDS	1301076	1302548	-		GGS 1324	98.98	SDE12394 07250	99.12
	SDEG 1352	CDS	1302779	1303270	-	*aroK*	GGS 1325	99.8	SDE12394 07255	99.39
	SDEG 1353	CDS	1303263	1304555	-		GGS 1326	100	SDE12394 07260	99.92
	SDEG 1354	CDS	1304653	1305609	-		GGS 1327	99.9	SDE12394 07265	100
	SDEG 1355	CDS	1305611	1306471	-	*map*	GGS 1328	99.77	SDE12394 07270	99.77
	SDEG 1356	CDS	1306488	1307783	-		GGS 1329	99.85	SDE12394 07275	99.92
	SDEG 1357	CDS	1307764	1308318	-		GGS 1330	99.28	SDE12394 07280	99.46
	SDEG 1358	CDS	1308567	1310138	-	*spg*	GGS 1331	99.07	SDE12394 07285	99.37

* BlastN scores were against each CDS of GGS_124 .

Product (assinged with GGS 124)
lactoylglutathione lyase
truncated laminin-binding surface protein
truncated laminin-binding surface protein
truncated laminin-binding surface protein
histidine triad protein
extracellular protein
transposase
ISSdy1, transposase OrfB
ISSag4, truncated transposase orfA
truncated hypothetical protein
periplasmic component of efflux system
ABC transporter ATP-binding protein
ABC transporter permease protein
two-component response regulator
two component system histidine kinase
immunogenic secreted protein
hypothetical protein
hypothetical protein
complement inhibitor protein
streptococcal C5a peptidase
truncated hypothetical protein
laminin binding protein
streptococcal histidine triad protein
hypothetical protein
CsbD-like protein
dipeptide transport ATP-binding protein
dipeptide transport ATP-binding protein
dipeptide transport system permease protein
dipeptide transport system permease protein
truncated dipeptide-binding protein
dipeptide-binding protein
truncated transposase
truncated transposase

UDP-N-acetylmuramoylpentapeptide-lysineN(6)-alan yltransferase/UDP-N-acetylmuramoylpentapeptide-lysineN(6)-
Na+/H+ antiporter
IS1562 transposase
truncated transposase
truncated transposase
Product (assinged with GGS 124)
hypothetical protein
putative PBP 5 synthesis repressor
shikimate kinase
3-phosphoshikimate 1-carboxyvinyltransferase
ribonuclease BN
methionine aminopeptidase
cytosolic protein containing multiple CBS domains
acetyltransferase GNAT family
immunoglobulin G-binding protein
