# Supplementary figures and images for: Evolutionary paths of streptococcal and staphylococcal superantigens
Source: BMC Genomics. 2012 Aug 17;13:404. doi: 10.1186/1471-2164-13-404 (PMC3538662; doi:10.1186/1471-2164-13-404)

0.100000

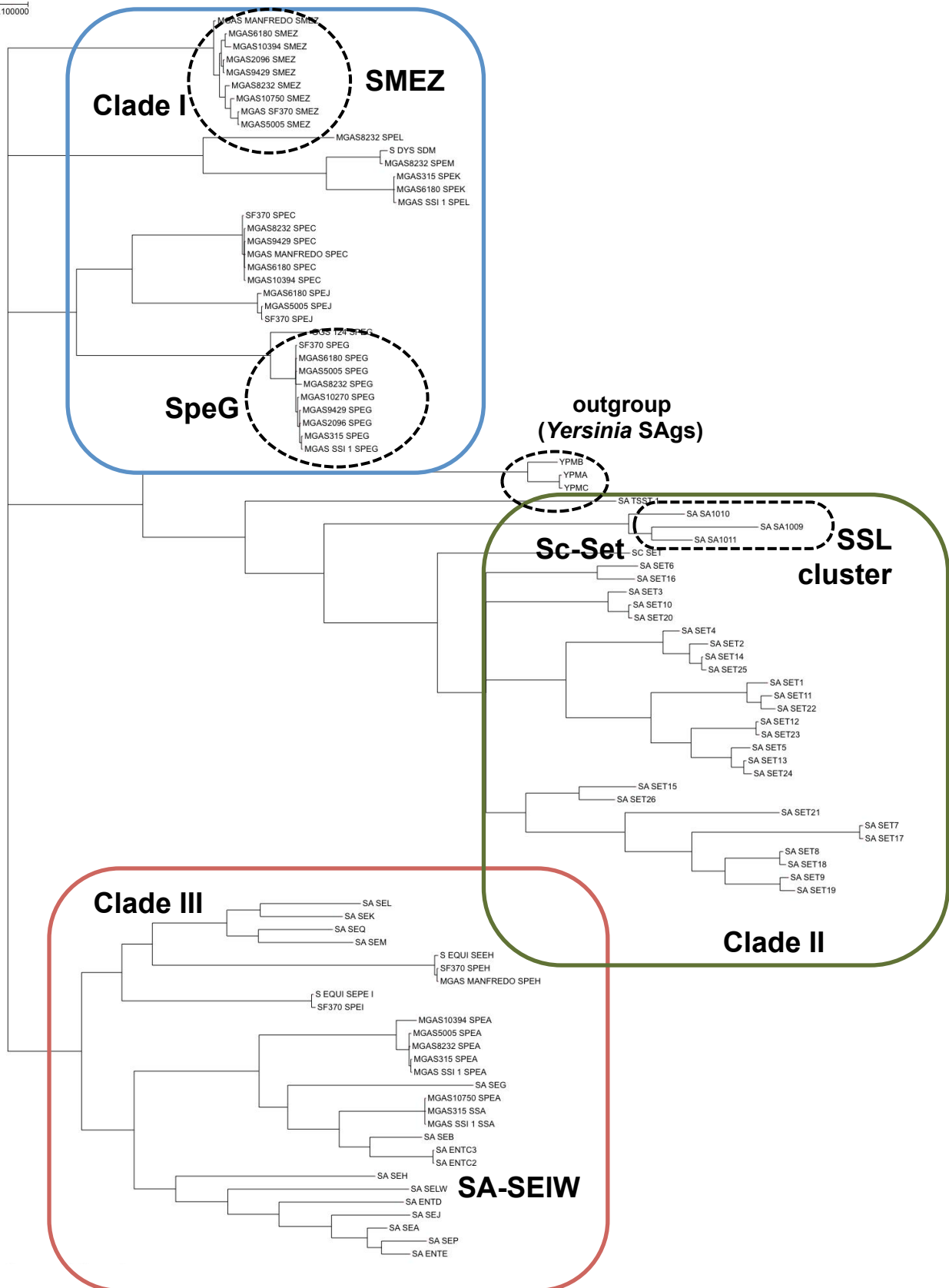

Supplement: Additional file 7 — Phylogenetic tree of streptococcal and staphylococcal SAgs and SSL amino acid sequences. The phylogenetic tree was constructed using the Bayesian MCMC method, with 100,000 generations and a mixed amino acid evolution model. The resultant potential scale reduction factor was 1.078. Essentially the same result was obtained by changing the number of generations and using the amino acid evolution model (data not shown). The nucleotide sequences used for alignment are shown in Additional file 9. The resulting phylogenetic tree was composed of three clades, with clade I including only streptococcal SAgs, clade II including only staphylococcal SSLs, and clade III including SAgs from both species. Orthologous gene products, including SpeG and SMEZ in clade I, SSL-like proteins in clade II and SElW in clade III, are emphasized. Yersinia SAgs (YPMA, YPMB, and YPMC) were also included as an outgroup. [file 1471-2164-13-404-S7.pdf]
