# Supplementary material for: Evolutionary paths of streptococcal and staphylococcal superantigens
Source: BMC Genomics. 2012 Aug 17;13:404. doi: 10.1186/1471-2164-13-404 (PMC3538662; doi:10.1186/1471-2164-13-404)
Supplement: Additional file 9 — List of Accession numbers for SAgs used in this study. [file 1471-2164-13-404-S9.doc]

**Additional file 9. List of Accession numbers for SAgs used in this study.**

| Species/strains | Name | Accesssion no.  (nucleotides) | Accesssion no.  (amino acids) |
| --- | --- | --- | --- |
| *S. dysgalactiae* subsp. *equisimilis* GGS_124 | SpeG | AP010935 | BAH82473 |
| *S. dysgalactiae subsp. dysgalactiae* | SDM | AB074529 | BAB93001 |
| *S. equi* subsp. *equi* 4047 | SEEH | FM204883 | AAF72809 |
|  | SEPE I | FM204883 | AAF72808 |
| *S*. *pyogenes* SF370 | SpeC | AE004092 | AAK33664 |
|  | SpeG | AE004092 | AAK33303 |
|  | SpeH | AE004092 | AAK33907 |
|  | SpeI | AE004092 | AAK33906 |
|  | SpeJ | AE004092 | AAK33456 |
|  | SMEZ | AE004092 | AAK34680 |
| *S*. *pyogenes* MGAS5005 | SpeA | CP000017 | AAZ51614 |
|  | SpeG | CP000017 | AAZ50801 |
|  | SpeJ | CP000017 | AAZ50974 |
|  | SMEZ | CP000017 | AAZ52320 |
| *S*. *pyogenes* SSI-1 | SpeA | BA000034 | BAC63655 |
|  | SpeG | BA000034 | BAC63256 |
|  | SpeL | BA000034 | BAC63752 |
|  | SSA | BA000034 | BAC64214 |
| *S*. *pyogenes* MGAS315 | SpeA | AE014074 | AAM79908 |
|  | SpeG | AE014074 | AAM78762 |
|  | SpeK | AE014074 | AAM79812 |
|  | SSA | AE014074 | AAM79527 |
| *S*. *pyogenes* MGAS10750 | SpeA | CP000262 | ABF38227 |
|  | SpeJ | CP000262 | ABF37571 |
|  | SMEZ | CP000262 | ABF38745 |
| *S*. *pyogenes* Manfredo | SpeC | AM295007 | CAM30589 |
|  | SpeH | AM295007 | CAM30348 |
|  | SMEZ | AM295007 | CAM30992 |
| *S*. *pyogenes* MGAS10394 | SpeA | CP000003 | AAT86876 |
|  | SpeC | CP000003 | AAT87331 |
|  | SMEZ | CP000003 | AAT87844 |
| *S*. *pyogenes* MGAS2096 | SpeA | CP000261 | ABF35653 |
|  | SpeG | CP000261 | ABF35247 |
|  | SMEZ | CP000261 | ABF36782 |
| *S*. *pyogenes* MGAS9429 | SpeC | CP000259 | ABF31781 |
|  | SpeG | CP000259 | ABF31372 |
|  | SMEZ | CP000259 | ABF32895 |
| *S*. *pyogenes* MGAS8232 | SpeA | AE009949 | AAL97141 |
|  | SpeC | AE009949 | AAL97445 |
|  | SpeG | AE009949 | AAL96995 |
|  | SpeL | AE009949 | AAL97848 |
|  | SpeM | AE009949 | AAL97849 |
|  | SMEZ | AE009949 | AAL98535 |
| *S*. *pyogenes* MGAS6180 | SpeC | CP000056 | AAX72082 |
|  | SpeG | CP000056 | AAX71294 |
|  | SpeK | CP000056 | AAX72333 |
|  | SpeJ | CP000056 | AAX71459 |
|  | SMEZ | CP000056 | AAX72797 |
| *Staphylococcus aureus* | SHE | AY345144 | CAI77677 |
|  | SEG | AY920260 | AAX11325 |
|  | SEB | AB479118 | AAL04126 |
|  | SEC3 | M28364.1 | AAA26624 |
|  | SEC2 | AIEQ01000005 | P34071 |
|  | SED | M28521 | P20723 |
|  | SEJ | AF053140 | AAC78590 |
|  | SEP | BA000018 | BAB43036 |
|  | SEA | CP001996 | AAP37183 |
|  | SEE | M21319.1 | P12993 |
|  | SEL | AF217235 | BAB58170 |
|  | SEK | GQ358928 | AAL04147 |
|  | SEQ | AF410775 | AAL04146 |
|  | SEM | EF551341 | ABQ42204 |
|  | TSST-1 | AY074881 | BAB58173 |
|  | SET-1 | AF188835 | CAG39450 |
|  | SET-2 | AJ938182 | CAI80071 |
|  | SET-3 | CP002110 | CAG39449 |
|  | SET-4 | BX571856 | CAG39452 |
|  | SET-5 | CP002110 | CAG39451 |
|  | SET-6 | FR714927 | BAB41610 |
|  | SET-7 | BA000017 | BAB41611 |
|  | SET-8 | BA000018 | BAB41612 |
|  | SET-9 | BA000018 | BAB41613 |
|  | SET-10 | BA000018 | BAB41614 |
|  | SET-11 | BA000018 | BAB41615 |
|  | SET-12 | BA000018 | BAB41616 |
|  | SET-13 | BA000018 | BAB41617 |
|  | SET-14 | BA000018 | BAB41618 |
|  | SET-15 | BA000018 | BAB41622 |
|  | SET-16 | BA000033 | BAB94247 |
|  | SET-17 | BA000033 | BAB94248 |
|  | SET-18 | BA000033 | BAB94249 |
|  | SET-19 | BA000033 | BAB94250 |
|  | SET-20 | BA000033 | BAB94251 |
|  | SET-21 | BA000033 | BAB94252 |
|  | SET-22 | BA000033 | BAB94253 |
|  | SET-23 | BA000033 | BAB94254 |
|  | SET-24 | BA000033 | BAB94255 |
|  | SET-25 | BA000033 | BAB94256 |
|  | SET-26 | BA000033 | BAB94259 |
|  | SA1009 | BA000018 | BAB42261 |
|  | SA1010 | BA000018 | BAB42262 |
|  | SA1011 | BA000018 | BAB42263 |
| (designated in this study) | SElW | CP000046 | BAB42694 |
| *S*. *carnosus* subsp. *carnosus* TM300 | Sc-Set | AM295250 | CAL27814 |
