# Supplementary material for: Evolutionary paths of streptococcal and staphylococcal superantigens
Source: BMC Genomics. 2012 Aug 17;13:404. doi: 10.1186/1471-2164-13-404 (PMC3538662; doi:10.1186/1471-2164-13-404)
Supplement: Additional file 10 — List of Accession numbers for SAgs used in this study. [file 1471-2164-13-404-S10.pdf]

Additional file 10. List of positions (bp) on each genome.

Figure 1	strain	position (bp)
	GGS_124	1944544-1903966
	SF370	164979-208255
	MGAS2096	171782-217290
	MGAS5005	165586-208866
	MGAS9429	179846-215477
	MGAS10270	171113-213081
	NZ131	169361-211323
	MGAS6180	170422-212388
	SSI-1	160008-193858
	MGAS315	166098-199948
	Manfredo	166807-201726
	MGAS8232	166178-201015
	MGAS10394	207062-240895
	MGAS10750	173587-208692
Figure 4	strain	position (bp)
	SF370	1654795-1680454
	MGAS5005	1651890-1678751
	MGAS2096	1669183-1697524
	MGAS9429	1645297-1673624
	MGAS6180	1682231-1709425
	SSI-1	1716393-1739618
	MGAS315	1722607-1745832
	Manfredo	1645959-1672446
	MGAS8232	1712675-1739147
	MGAS10394	1702587-1729183
	MGAS10750	1737490-1765283
	MGAS10270	1708676-1736550
	NZ131	1619784-1652050
	GGS_124	182262-157414 (for *flaR* region)
		943764-918282 (for *dpp* region)
	RE378	197672-173072 (for *flaR* region)
		977063-951518 (for *dpp* region)
	ATCC 12394	185972-157414 (for *flaR* region)
		1035865-1010250 (for *dpp* region)
Figure 6	strain	position (bp)
	COL	1659847-1711873
	JH1	1731441-1782697
	JH9	1731567-1782823
	MRSA252	1717536-1768502
	MSSA476	1632090-1683170
	Mu3	1684599-1735855
	Mu50	1683199-1734454
	MW2	1652380-1703518
	N315	1606787-1658042
	NCTC8325	1585886-1637913
	newman	1642685-1694712
	RF122	1561945-1613938
	TCH1516	1683274-1735300
	FPR3757	1682684-1732829
Figure 8	strain	position (bp)
	COL	1159176-1208843

	JH1	1243965-1293709
	JH9	1244091-1293835
	MRSA252	1160194-1210781
	MSSA476	1148225-1197704
	Mu3	1197242-1246986
	Mu50	1195841-1245586
	MW2	1119439-1169031
	N315	1119513-1169257
	NCTC8325	1055514-1105077
	newman	1155367-1205034
	RF122	1086900-1136250
	TCH1516	1149585-1198971
	FPR3757	1135638-1185024
Additional file 8	strain	position (bp)
	COL	1276590-1326336
	JH1	1361645-1411363
	JH9	1361771-1411489
	MRSA252	1279035-1328747
	MSSA476	1265829-1315541
	Mu3	1314926-1364638
	Mu50	1313526-1363238
	MW2	1237328-1287046
	NCTC8325	1172818-1222562
	newman	1272775-1322518
	RF122	1202213-1252183
	TCH1516	1266711-1316479
	FPR3757	1252764-1302532
	N315	1237197-1286909
	TM300 (*S. carnosus*)	886070-935300
